# Do primary and secondary care doctors have a different experience and perception of cross-level clinical coordination? Results of a cross-sectional study in the Catalan National Health System (Spain)

**DOI:** 10.1186/s12875-020-01207-9

**Published:** 2020-07-08

**Authors:** Laura Esteve-Matalí, Ingrid Vargas, Elvira Sánchez, Isabel Ramon, Pere Plaja, María-Luisa Vázquez

**Affiliations:** 1 Health Policy and Health Services Research Group, Health Policy Research Unit, Consortium for Health Care and Social Services of Catalonia, Barcelona, Spain; 2 Department for Paediatrics, Obstetrics and Gynaecology, Preventive Medicine, Universitat Autònoma de Barcelona, Catalonia, Spain; 3 Grup de Recerca en Serveis Sanitaris i Resultats en Salut (GRESSIRES), Serveis de Salut Integrats Baix Empordà (SSIBE), Palamós, Spain; 4 Consorci Hospitalari de Vic, Vic, Spain; 5 Fundació Salut Empordà, Figueres, Spain

**Keywords:** Clinical coordination across care levels, Integrated care, Health systems research, Questionnaire

## Abstract

**Background:**

Clinical coordination across care levels is a priority for health systems around the world, especially for those based on primary health care. The aim of this study is to analyse the degree of clinical information and clinical management coordination across healthcare levels in the Catalan national health system experienced by primary (PC) and secondary care (SC) doctors and explore the associated factors.

**Methods:**

Cross-sectional study based on an online survey using the self-administered questionnaire COORDENA-CAT. Data collection: October–December 2017. Study population: PC and SC (acute and long term) doctors of the Catalan national health system. Participation rate was 21%, with a sample of 3308 doctors. Outcome variables: cross-level clinical information coordination, clinical management coordination, and perception of cross-level coordination within the area. Explanatory variables: socio-demographic, employment characteristics, attitude towards job, type of area (according to type of hospital and management), interactional factors, organizational factors and knowledge of existing coordination mechanisms. Stratification variable: level of care. Descriptive and multivariate analysis by logistic regression.

**Results:**

The degree of clinical coordination experienced across levels of care was high for both PC and SC doctors, although PC doctors experienced greater exchange and use of information and SC doctors experienced greater consistency of care. However, only 32.13% of PC and 35.72% of SC doctors found that patient care was coordinated across care levels within their area. In both levels of care, knowing the doctors of the other level, working in an area where the same entity manages SC and majority of PC, and holding joint clinical case conferences were factors positively associated with perceiving high levels of clinical coordination. Other associated factors were specific to the care level, such as being informed of a patient’s discharge from hospital for PC doctors, or trusting in the clinical skills of the other care level for SC doctors.

**Conclusions:**

Interactional and organizational factors are positively associated with perceiving high levels of clinical coordination. Introducing policies to enhance such factors can foster clinical coordination between different health care levels. The COORDENA questionnaire allows us to identify fields for improvement in clinical coordination.

## Background

Over recent decades, clinical coordination has become a challenge as well as a priority for health systems around the world, especially for those based on primary health care, such as the Spanish national health system (NHS). High specialization, rapid technological innovations and new forms of organizing health services imply the involvement of multiple services and providers in patient care, a situation which particularly affects chronic patients or with comorbidities [1, 2].

Better coordination between levels of care is expected to improve the quality of health services and increase efficiency, effectiveness and access to health services [3–7]. Given the importance of coordination, many strategies at micro-, meso- and macro-level have been developed in order to improve collaboration between health care providers [4, 8, 9].

Despite the interest and increase in publications, research on clinical coordination is limited by a lack of consensus on definitions across disciplines [10]. This study is based on a conceptual framework on care coordination across care levels that addresses the different types and dimensions of clinical coordination as well as its related factors [11]. Clinical coordination is defined as the harmonious connection of the different health services needed to provide care to a patient throughout the care continuum in order to achieve a common objective without conflicts [12, 13]. Two interrelated types can be distinguished [14, 15]:

- Clinical information coordination, which refers to the appropriate transfer and use of patient clinical information between providers.
- Clinical management coordination, which refers to the provision of care in a sequential and complementary way by the different services and healthcare levels involved. It encompasses three dimensions: consistency of care, adequate patient follow-up and accessibility between levels of care.

Clinical coordination is an important component of care coordination, along with administrative coordination, or coordination of patient access across the continuum of services according to their needs [16]. Care coordination requires not only the coordination of care activities (clinical coordination) but also the coordination of key support functions and activities (such as financial management, strategic planning, human resources policies, etc.) across the operating units that make up the health services network [17].

Available qualitative and quantitative studies have identified different types of factors that can influence clinical coordination: organizational factors, such as the management model or the availability of coordination mechanisms [18–21] and factors related to professionals, such as attitude towards coordination or knowing the doctors of the other care level [18–21]. Clinical coordination can be analysed through service-based indicators or from the perspective of health professionals using quantitative or qualitative methods [12].

Most of the evidence available on clinical coordination at both the international and national level is limited to a specific pathology, focused on only one dimension of coordination (mainly information transfer across levels), or on one coordination mechanism or strategy (mainly electronic medical record) [22–25]. In general, the main clinical coordination problem studied is poor information transfer and difficulties in communicating with the other care level [26, 27]. Although the perspective of doctors is relevant to the design of strategies to improve clinical coordination [28], there are few studies available based on surveys of doctors and they rarely contemplate the factors associated with coordination [29, 30]. Moreover, doctors from different levels of care, who are expected to collaborate on the patient care, might experience clinical coordination differently, according to their needs and expectations, and therefore offer a different perspective on the same phenomenon. Quantitative studies that comprehensively analyse clinical coordination, its determinants and the contribution of coordination mechanisms from the perspective of primary and secondary care doctors are extremely scarce.

In the Spanish NHS, in which primary health care acts as the gate-keeper and coordinator of patient care across the different healthcare levels, clinical coordination is still an unresolved issue [28, 31, 32], so it is crucial to foster collaboration between doctors from different levels of care. In the Spanish region of Catalonia, the NHS is characterized by a split of the financing and provision functions. The provision of services is the responsibility of a number of contracted providers; a large public entity, the Catalan Health Institute (Institut Català de la Salut), and a number of public consortia, municipal foundations and some private foundations (mostly non-profit but also some for profit), which make up the Integrated Healthcare System for Public Use (Sistema Sanitari Integral d’Utilització Pública de Catalunya) [33]. This diversity has generated a variety of management models of primary (PC) and secondary care (SC) providers across the healthcare areas of the national health system. According to the degree of management integration of PC/SC providers, the healthcare areas can be classified in: 1) areas in which a single entity manages SC and majority of PC centres (joint management); 2) one entity manages SC and some primary care centres, while the rest are managed by other entities (partially integrated); 3) different entities manage SC and PC (non-integrated) [34, 35]. The coexistence of management models offers an interesting landscape for health services research.

Clinical coordination in Catalonia has been previously analysed by means of qualitative studies [18, 20, 21, 36] which showed limits in cross-level coordination related to factors regarding the system, health professionals or organizational factors. More comprehensive evidence is needed on clinical coordination in its different types and dimensions, as well as a greater focus on the general population, from the perspective of primary and secondary care doctors [37].

## Methods

### Aim

The aim of this study, which forms part of a wider research project [37], is to analyse the degree of clinical information and clinical management coordination across healthcare levels in the Catalan NHS experienced by primary and secondary care doctors and explore the associated factors.

### Study design and areas of study

A cross-sectional study was carried out based on an online survey of primary care (PC) and secondary care (SC) doctors of the Catalan NHS by self-administration of the questionnaire COORDENA-CAT [19]. The areas of study were defined on the basis of primary healthcare areas and their referral hospitals (acute and long-term care).

### Study population and sample

The study population consisted of PC and SC doctors that had been working for at least one year in the organization, had direct contact with patients and whose daily practice involved contact with doctors of the other care level (i.e. through the patient referral process). A total of 15,813 doctors from 41 areas of the public national health system in Catalonia were invited to participate. The final sample was 3308 doctors (1141 from PC and 2167 from SC) from 32 healthcare areas. The participation rate was 21%.

### Questionnaire

The COORDENA questionnaire was originally designed in Latin America, based on the theoretical framework underlying this study [11] and an extent scientific literature review, and it was tested, piloted and validated. The COORDENA-CAT questionnaire, the online version of the COORDENA questionnaire used in this study, was adapted to the Catalan NHS context and validated [37]. It is divided into three main parts: the first, which is the focus of this paper, measures doctors’ experience of clinical information and clinical management coordination across care levels and their general perception of coordination within the area, by means of a Likert scale and one open-ended question on their reasons for that perception. The second measures their knowledge and experience in the use of coordination mechanisms across care levels, and the third refers to the factors that might influence clinical coordination.

### Data collection

Data was collected from October to December 2017. Invitations were sent to the institutional doctors’ e-mails, by their own institutions. Each invitation contained a link, randomly generated that gave anonymous access to the questionnaire. In addition to the invitation sent by e-mail, and following reminders after one and two weeks, doctor participation was encouraged using different strategies such as informative sessions in their centres, posters and news posts on the intranet of their institutions, strategies that were tested on the pilot study of the questionnaire [37].

### Variables

The outcome variables of clinical coordination across levels were a) *coordination of clinical information between levels*: transfer and use of information (three items); b) *coordination of clinical management*: consistency of care (four items), adequate follow up (four items) and accessibility between levels (three items); and c) *general perception of the coordination of patient care in the area* (one item) and the underlying reasons. Outcome variables are described in Tables 3 and 4. Level of care was a stratification variable.

The explanatory variables used to explore factors associated with perceiving high levels of clinical coordination were a) *sociodemographic*: sex, age, country of birth and medical speciality; b) *employment characteristics*: years working as a doctor, years working in the organization and type of contract; c) *attitude towards job:* satisfaction with their job in the organization d) *type of area*: type of management of PC and SC and type of hospital; e) *interactional factors between doctors*: knowing the doctors of the other care level, trusting in their clinical skills, perceiving that they influence the practice of doctors of the other level, and identifying the PC doctor as the coordinator of patient care across levels; f) *organizational factors*: perceiving that the organization’s management facilitates coordination, existence of objectives aimed at coordination in the organization, having enough time to dedicate to coordination during working hours, and visits of secondary care doctors to primary care centres; g) *knowledge of existing coordination mechanisms in the area*: shared electronic medical record (EMR) of the organization, joint clinical case conferences between primary and secondary care doctors, virtual consultations through EMR or e-mail, consultations via phone, shared protocols, care pathways or clinical guidelines, case managers or liaison nurses, and informing primary care doctors when their patients are discharged from hospital. Explanatory variables are described in Tables 1 and 2.

**Table 1:** Description of the sample by level of care: sociodemographic, employment characteristics and type of area

	Total	PC	SC	
	(*N* = 3308)	(*N* = 1141)	(*N* = 2167)	
	n (%)	n (%)	n (%)	*p*
***Sociodemographic characteristics***				
Sex				***< 0.001***
Male	1214 (42.12)	307 (31.01)	907 (47.94)	
Female	1668 (57.88)	683 (68.99)	985 (52.06)	
Age				***< 0.001***
25–40 years	701 (25.46)	180 (18.97)	521 (28.88)	
41–55 years	1278 (46.42)	486 (51.21)	792 (43.90)	
56–70 years	774 (28.11)	283 (29.82)	491 (27.22)	
Country of birth				*0.345*
Spain	2469 (87.37)	858 (88.18)	1611 (86.94)	
Other	357 (12.63)	115 (11.82)	242 (13.06)	
Medical speciality				***< 0.001***
Clinical speciality	2163 (78.37)	954 (98.76)*	1209 (67.39)	
Surgical speciality	265 (9.60)	0 (0)	265 (14.77)	
Medical and surgical speciality	332 (12.03)	12 (1.24)	320 (17.84)	
***Employment characteristics***				
Years working as a doctor				***< 0.001***
0 to 10 years	378 (13.54)	88 (9.11)	290 (15.89)	
11 to 20 years	929 (33.29)	329 (34.06)	600 (32.88)	
21 to 30 years	805 (28.84)	318 (32.92)	487 (26.68)	
31 to 45 years	679 (24.33)	231 (23.91)	448 (24.55)	
Years working in the organization				***< 0.001***
1 to 5 years	406 (14.62)	78 (8,13)	328 (18.05)	
6 to 15 years	972 (35)	297 (30.94)	675 (37.15)	
16 to 25 years	769 (27.69)	338 (35.21)	431 (23.72)	
26 to 45 years	630 (22.69)	247 (25.73)	383 (21.08)	
Type of contract a)				***< 0.001***
Permanent	2630 (90.94)	965 (96.31)	1665 (88.10)	
Temporary	262 (9.06)	37 (3.69)	225 (11.90)	
Type of contract b)				*0.233*
Full-time	2660 (91.88)	929 (92.71)	1731 (91.44)	
Part-time	235 (8.12)	73 (7.29)	162 (8.56)	
***Attitude towards job***				
Satisfaction with the job in the organization				***0.008***
Yes	2193 (84.80)	741 (82.24)	1452 (86.17)	
No	393 (15.20)	160 (17.76)	233 (13.83)	
***Type of area***				
Area according to type of management of PC and SC				***0.001***
One entity manages SC and majority of PC	1457 (44.04)	477 (41.81)	980 (45.22)	
One entity manages SC and minority of PC	892 (26.96)	354 (31.03)	538 (24.83)	
Different entities manage SC and PC	959 (28.99)	310 (27.17)	649 (29.95)	
Area according to type of hospital				***< 0.001***
Local or regional hospitals	1857 (56.14)	770 (67.48)	1087 (50.16)	
Regional high-resolution hospitals	810 (24.49)	222 (19.46)	588 (27.13)	
High-technology hospitals	641 (19.38)	149 (13.06)	492 (22.70)	

* Family doctors

**Table 2:** Interactional and organizational factors and knowledge of coordination mechanisms, by level of care

	Total	PC	SC	
	(N = 3308)	(N = 1141)	(N = 2167)	
	n (%)	n (%)	n (%)	*p*
***Interactional factors between doctors***				
I know the doctors of the other care level who see my patients personally^a^	1103 (39.06)	442 (44.02)	661 (36.32)	***< 0.001***
I trust in the clinical skills of the doctors of the other level who see my patients^a^	2429 (88.62)	971 (97.20)	1458 (83.70)	***< 0.001***
My daily practice influences the practice of the doctors of the other level^a^	1834 (78.61)	554 (66.19)	1280 (85.56)	***< 0.001***
In practice, primary care doctors are responsible for coordinating the patient on their way through the different levels of care^a^	2189 (81.92)	950 (95.19)	1239 (74.01)	***< 0.001***
***Organizational factors***				
My organization’s management facilitates coordination between primary and secondary care doctors^a^	1492 (59.18)	665 (67.44)	827 (53.88)	***< 0.001***
My organization sets objectives that are aimed at coordination between care levels^a^	1370 (55.20)	540 (56.72)	830 (54.25)	*0.228*
The time I can dedicate to coordinating with doctors of the other level during my working day is sufficient ^a^	380 (13.90)	136 (13.67)	244 (14.03)	*0.792*
As a secondary care doctor, do you do patient consultations in a primary care centre?^a^			386 (21.44)	
***Knowledge of the coordination mechanisms available in the area***				
The electronic medical records that you use in your centre allows you to share information between primary and secondary (hospital, long-term) care^b^	2623 (89.92)	918 (87.60)	1705 (91.23)	***0.002***
In your centre, you can hold virtual consultations through the EMR between primary and secondary care doctors^b^	1795 (72.55)	841 (83.18)	954 (65.21)	***< 0.001***
In your centre, you can hold consultations via e-mail between primary and secondary care doctors^b^	1885 (75.98)	792 (79.52)	1093 (73.60)	***0.001***
In your centre, you can hold consultations via phone between primary and secondary care doctors^b^	2085 (79.73)	700 (71.72)	1385 (84.50)	***< 0.001***
In your centre, you hold joint clinical case conferences between primary and secondary care doctors for the discussion of cases^b^	1226 (43.50)	696 (66.60)	530 (29.91)	***< 0.001***
In your centre, you have at your disposal shared protocols, care pathways or clinical practice guidelines between primary and secondary care^b^	1847 (74.99)	940 (92.16)	907 (62.86)	***< 0.001***
There are case managers or liaison nurses at your centre^b^	2031 (80.28)	876 (87.43)	1155 (75.59)	***< 0.001***
Primary care doctors are informed when their patients are discharged from the hospital^a^	1105 (43.61)	677 (64.05)	428 (28.98)	***< 0.001***

^a^ Results correspond to the categories *always* and *very often*

^b^ Results correspond to the category *yes*

### Analysis

Univariate and bivariate analyses were performed to describe the experience and perception of clinical coordination across levels (outcome variables) and the potentially associated factors (explanatory variables), stratified by level of care. Chi-square tests were performed to determine statistically significant differences between PC and SC doctors.

Logistic regression models for each level of care were generated in order to explore the factors associated with the general perception of high coordination across care levels within the area. Robust covariance adjustments – employing type of area according to the type of management of PC and SC – were used to account for correlated observations due to clustering. Raw and adjusted *odds ratios* (OR) at the 95% confidence interval (CI 95%) were calculated. To reach the final models, the explanatory variables were introduced by groups on a forward stepwise way, keeping the significant and theoretically relevant ones in the model: first, sociodemographic; second, employment characteristics; third, attitude towards job; fourth, type of area; fifth, doctors’ interactional factors; sixth, organizational factors and lastly, knowledge of existing coordination mechanisms in the area. This allowed us to ascertain the impact of different types of variables on adjusting the model. Multicollinearity between the explanatory variables was assessed by a correlation matrix as well as by testing the variance inflation factor (VIF), which was found to be insignificant (below 1.5) in both final models. The fitness of the models was assessed with the Hosmer-Lemeshow test, which gave *p*-values over 0.05 indicating the goodness of fit of the models. The statistical software used was STATA 15.

Finally, a content analysis was performed for an open-ended question on reasons for the general perception of clinical coordination between care levels within the area. The answers were coded and classified into categories. Frequencies were calculated stratified by PC and SC doctors.

## Results

### Characteristics of the sample

With regard to sociodemographic characteristics, the majority of doctors in the sample in both care levels were women (68.99% PC; 52.05% SC), and the highest proportion were 41–55 years of age (51.21% PC; 43.90% SC). Most were trained in a medical speciality: almost all PC doctors (98.76%), as family doctors, and 67.30% of SC (Table 1).

In terms of employment characteristics, the highest proportion of doctors had 11 to 20 years’ work experience (34.06% PC; 32.88% SC), and a little less experience working in the organization, 6 to 15 years (30.94% PC; 37.15% SC). The majority had a permanent contract (96.31% PC; 88.10% SC) (Table 1).

With regard to attitude, most doctors were satisfied with their job, but SC doctors (86.17%) slightly more so than PC doctors (82.24%). With respect to the type of area, 41.81% of PC and 45.22% of SC doctors worked in an organization where the same entity manages SC and the majority of PC; and 67.48% of PC and 50.16% of SC doctors worked in areas with local and regional hospitals (Table 1).

In terms of interactional factors, less than half (44.02% PC; 36.32% SC) reported knowing the doctors of the other care level, but the great majority (97.20% PC; 83.70% SC) reported trusting in their clinical skills. Finally, the majority, but fewer PC (66.19%) than SC (85.56%) doctors, perceived that the care they provide influences the practice of the doctors of the other level (Table 2).

Regarding organizational factors, 67.44% of PC and 53.88% of SC doctors found that their organization’s management facilitates coordination across levels (Table 2). The degree of knowledge of the cross-level coordination mechanisms available in the area was relatively high, especially for the shared EMR of the centre (87.60% PC; 91.23% SC), although percentages differed between PC and SC doctors. The least accessible coordination mechanism among both PC and SC doctors was joint clinical case conferences (66.60% PC; 29.91% SC). There was a notable difference between care levels in access to standardization mechanisms such as shared protocols, care pathways or clinical practice guidelines (92.16% PC; 62.86% SC) (Table 2).

### Primary and secondary care doctors’ experience of clinical information and clinical management coordination between levels

With regard to the coordination of clinical information across levels of care, the majority of doctors from both care levels reported frequent exchange and use of the information required for patient care, with higher frequency among PC doctors. A higher proportion of PC doctors reported that the patient’s clinical information is usually shared between levels of care (70.09% PC; 59.39% SC); that the information shared is as required for the care of the patient (76.92% PC; 69.56% SC) and that this information is frequently used (84.90% PC; 80.06%SC) (Table 3).

**Table 3:** Primary and secondary care doctors’ experience of high levels of clinical coordination

	Total	PC	SC	
	(N = 3308)	(N = 1141)	(N = 2167)	
	n (%)	n (%)	n (%)	*p*
***Coordination of clinical information between levels of care***				
Primary and secondary care doctors share information on the care of patients we have in common^a^	1959 (63.11)	757 (70.09)	1202 (59.39)	***< 0.001***
The information we share is as required for the care of these patients^a^	2224 (72.14)	830 (76.92)	1394 (69.56)	***< 0.001***
Primary and secondary care doctors use the information that we share^a^	2489 (81.77)	911 (84.90)	1578 (80.06)	***0.001***
***Coordination of clinical management: Consistency of care between levels***				
We agree with the treatments prescribed or directions given to the patients by doctors of the other level^a^	2357 (77.99)	792 (74.16)	1565 (80.09)	***< 0.001***
There are no contraindications and/or duplications in the treatments prescribed by primary and secondary care doctors^a^	2100 (69.54)	652 (61.05)	1448 (74.18)	***< 0.001***
Primary and secondary care doctors establish a treatment plan together for patients that require this^a^	422 (13.82)	129 (11.99)	293 (14.82)	***0.030***
We do not repeat the tests that doctors have already carried out at the other level (analysis, imaging)^a^	2182 (71.45)	768 (71.98)	1414 (71.16)	*0.635*
***Coordination of clinical management: Adequate follow-up between levels***				
Primary care doctors refer the patients to secondary care when appropriate^a^	2493 (83.13)	1060 (99.07)	1433 (74.29)	***< 0.001***
Secondary care doctors send the patients back to primary care for follow-up when appropriate^a^	2484 (83.55)	811 (76.65)	1673 (87.36)	***< 0.001***
Secondary care doctors make recommendations to the primary care doctor on the follow-up of patients (diagnosis, treatment, other guidelines)^a^	1780 (59.10)	421 (39.38)	1359 (69.94)	***< 0.001***
Primary care doctors resolve any queries on the follow-up of patients with the secondary care doctors^a^	1249 (42.63)	588 (55.00)	661 (35.52)	***< 0.001***
***Coordination of clinical management: Accessibility between levels of care***				
On being referred in the standard way to secondary care, the patient does not wait a long time to be seen^a^	611 (21.43)	17 (1.58)	594 (33.43)	***< 0.001***
On being referred urgently to secondary care, the patient does not wait a long time to be seen^a^	1440 (49.33)	189 (17.58)	1251 (69.15)	***< 0.001***
On being sent back to primary care, the patient does not wait a long time to be seen^a^	1540 (76.24)	870 (81.84)	670 (30.91)	***< 0.001***

^a^ Results correspond to the categories *always* and *very often*

The three dimensions of clinical management coordination showed relatively high frequencies in both care levels, with some differences (Table 3).

Regarding consistency of care, most doctors reported usually agreeing with the treatments prescribed by doctors of the other care level (74.16% PC; 80.09% SC) and not experiencing contradictions or duplications in the treatments prescribed by the other care level (61.05% PC; 74.18% SC). However, only 11.99% of PC and 14.82% of SC doctors reported establishing a treatment plan together for patients who require it.

For patient follow-up between levels, as expected, more PC doctors (99.07% PC; 74.29% SC) reported that PC doctors usually refer their patients to secondary care when appropriate and, vice versa, more SC doctors (87.36% SC; 76.65% PC) reported that SC doctors refer the patients back to primary care when appropriate. Similarly, a smaller proportion of PC doctors (39.38% PC; 69.94% SC) reported that SC doctors make recommendations to PC doctors, while a higher proportion of PC doctors (55% PC; 35.52% SC) reported that PC doctors contact SC doctors to resolve queries on the follow-up of patients.

Lastly, in terms of accessibility across levels of care, a notable minority of PC doctors reported that the patient does not usually have to wait long for an appointment upon standard (1.58% PC; 33.43% SC) or urgent (17.58% PC; 69.15% SC) referral to SC. Conversely, 81.84% of PC doctors and 30.91% of SC doctors reported that the patient does not have to wait long to be seen on being sent back to PC. However, it should be stressed that for the latter item, around 50% of SC doctors responded “I don’t know”, this being the only item in which this answer category was chosen with notable frequency.

### Primary and secondary care doctors’ perception of clinical coordination between levels in the area and underlying reasons

**Fig. 1:**
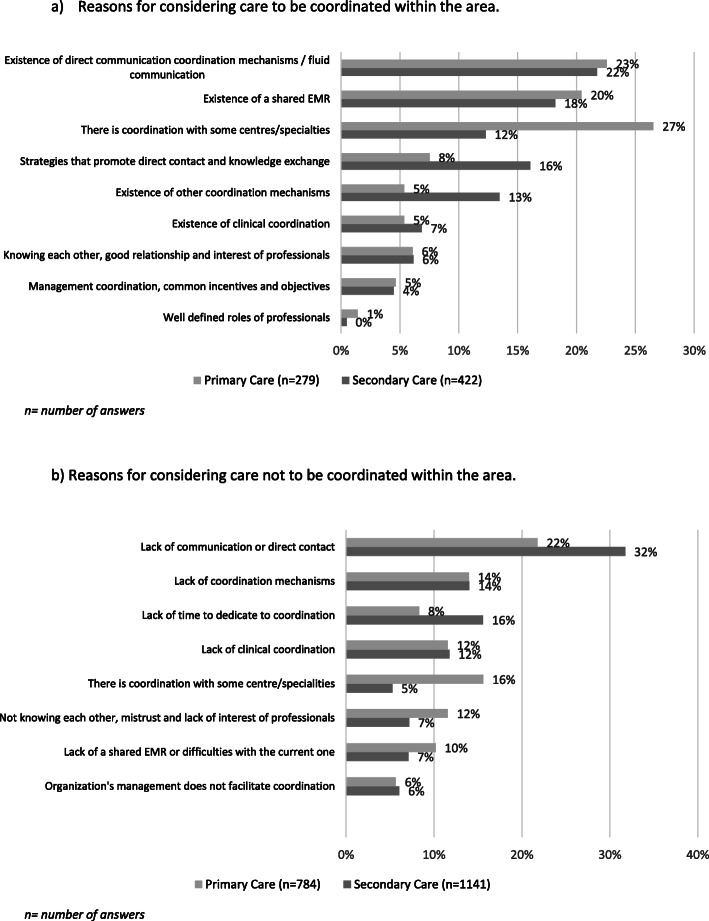
Reasons for the perception of clinical coordination within the area, by level of care. a) Reasons for considering care to be coordinated within the area. b) Reasons for considering care not to be coordinated within the area

Regarding the general perception of coordination across healthcare levels, only around a third of doctors (32.13% PC; 35.72% SC) reported that patient care is usually coordinated between primary and secondary care within their area (Table 4). The results of the open-ended question showed that reasons for this perception were similar among doctors of both care levels (Fig. 1). The most frequent reasons given for perceiving high coordination (Fig. 1a) were that there was fluid communication and direct communication mechanisms (phone, e-mail or virtual consultations; 23% PC; 22% SC); shared EMR (20% PC; 18% SC); strategies that promote direct contact and knowledge exchange (such as joint clinical case conferences; 8% PC; 16% SC) and coordination with some centres/specialities (27% PC; 12% SC). Accordingly, the most frequent reasons given for considering coordination to be poor (Fig. 1b) were related to a lack of communication or direct contact (22% PC; 32% SC) and a lack of coordination mechanisms (14% for both levels). The reasons included limited time to dedicate to coordination (8% PC; 16% SC).

**Table 4:** Primary and secondary care doctors’ general perception of high levels of coordination between healthcare levels

	Total	PC	SC	
	(N = 3308)	(N = 1141)	(N = 2167)	
	n (%)	n (%)	n (%)	*p*
***General perception of coordination between healthcare levels***				
I think that in this area patient care is coordinated between primary and secondary care doctors^a^	1012 (34.43)	339 (32.13)	673 (35.72)	***0.049***

^a^ Results correspond to the categories *always* and *very often*

### Factors associated to primary and secondary care doctor’s perception of high patient care coordination between levels

The item on general perception of the coordination of patient care in the area, “I think that in this area patient care is coordinated between primary and secondary care doctors”, was used to explore the associated factors (Table 5), showing some concordance but also some differences between PC and SC doctors. Factors positively associated with perceiving high levels of coordination for both PC and SC doctors were interactional: knowing the doctors of the other level, perceiving that their own practice influences the other level; and organizational: perceiving that the organization’s management facilitates coordination, working in an area where one entity manages SC and the majority of PC, and having access to joint clinical case conferences.

**Table 5:** Factors associated with perception of high levels of clinical coordination, by level of care

*Factors*	Primary Care		Secondary Care	
	Raw OR (95% IC)	Adj. OR (95% IC)	Raw OR (95% IC)	Adj. OR (95% IC)
Sex				
*Female*	1	1	1	1
*Male*	1.04 (0.78–1.39)	1.02 (0.69–1.50)	0.94 (0.77–1.14)	1.12 (0.82–1.54)
Country of birth				
*Spain*	**0.63 (0.42–0.95)**	0.85 (0.44–1.62)	1.21 (0.90–1.64)	1.19 (0.73–1.94)
*Other*	1	1	1	1
Years working as a doctor				
*0 to 10 years*	1	1	1	1
*11 to 20 years*	0.80 (0.49–1.32)	0.82 (0.31–2.20)	**0.62 (0.46–0.83)**	**0.49 (0.27–0.89)**
*21 to 30 years*	0.79 (0.48–1.30)	0.83 (0.32–2.13)	0.92 (0.68–1.25)	0.82 (0.43–1.57)
*31 to 45 years*	0.74 (0.44–1.24)	0.62 (0.24–1.60)	**0.63 (0.58–0.94**)	**0.50 (0.32–0.80)**
Satisfaction with the job in the organization				
*Yes*	**3.62 (2.25–5.83)**	**2.59 (2.09–3.21)**	**4.15 (2.78–6.19)**	
*No*	1	1	1	
I know the doctors of the other care level who see my patients personally				
*Often/Always*	**3.51 (2.66–4.64)**	**2.22 (1.58–3.10)**	**3.79 (3.08–4.67)**	**2.67 (1.64–4.43)**
*Rarely/Never*	1	1	1	1
I trust in the clinical skills of the doctors of the other level who see my patients				
*Often/Always*	2.84 (0.98–8.29)		**3.57 (2.54–5.01)**	**1.61 (1.18–2.19)**
*Rarely/Never*	1		1	1
My daily practice influences the practice of the doctors of the other level				
*Often/Always*	**2.30 (1.64–3.22)**	**1.72 (1.29–2.30)**	**3.06 (2.11–4.43)**	**2.08 (1.36–3.18)**
*Rarely/Never*	1	1	1	1
My organization’s management facilitates coordination between primary and secondary care doctors				
*Often/Always*	**4.43 (3.12–6.30)**	**2.56 (1.80–3.63)**	**5.15 (4.07–6.52)**	**3.59 (2.36–5.46)**
*Rarely/Never*	1	1	1	1
The time I can dedicate to coordinating with doctors of the other level during my working day is sufficient				
*Often/Always*	**1.87 (1.28–2.71)**		**3.97 (2.97–5.31)**	**2.20 (1.90–2.56)**
*Rarely/Never*	1		1	1
Area according to type of management of PC and SC				
*One entity manages SC and majority of PC*	1	1	1	1
*One entity manages SC and minority of PC*	**0.73 (0.54–0.99)**	**0.76 (0.71–0.82)**	**0.77 (0.61–0.98)**	**0.78 (0.70–0.87)**
*Different entities manage SC and PC*	**0.70 (0.51–0.97)**	**0.64 (0.58–0.72)**	1.17 (0.94–1.46)	1.05 (0.95–1.16)
Area according to type of hospital				
*Local and regional hospitals*	1	1	1	1
*Regional high-resolution hospitals*	1.14 (0.82–1.58)	1.15 (0.90–1.47)	1.19 (0.96–1.48)	1.14 (0.56–2.34)
*High-technology hospitals*	0.75 (0.49–1.13)	**0.54 (0.49–0.60)**	**0.78 (0.61–0.99)**	1.03 (0.69–1.55)
Joint clinical case conferences between primary and secondary care doctors for the discussion of cases				
*Yes*	**2.46 (1.81–3.34)**	**1.97 (1.32–2.94)**	**3.26 (2.63–4.05)**	**1.50 (1.39–1.62)**
*No*	1	1	1	1
Shared protocols, care pathways or clinical practice guidelines between primary and secondary care				
*Yes*	**3.99 (1.97–8.11)**		**3.77 (2.91–4.88)**	**2.42 (1.63–3.58)**
*No*	1		1	1
Primary care doctors are informed when their patients are discharged from the hospital				
*Often/Always*	**3.12 (2.30–4.25)**	**3.14 (2.53–3.89)**	**2.52 (1.99–3.19)**	
*Rarely/Never*	1	1	1	

In the specific case of PC doctors, other factors found to be associated with perceiving high coordination were job-related attitude: being satisfied with their job in the organization; and organizational: working in areas with local/regional hospitals, and being informed of their patients’ hospital discharge. Specifically for SC doctors, further associated factors were: less work experience as a doctor, trusting in the clinical skills of PC doctors, having enough time for coordination during working hours, and access to shared protocols, pathways or clinical practice guidelines (Table 5).

## Discussion

This study is the first attempt to comprehensively analyse clinical coordination between care levels in a national health system, taking into account the different types and dimensions of clinical coordination and the experience of primary and secondary care doctors, allowing us to analyse critical elements for improvement. The degree of clinical coordination experienced across levels of care was relatively high, although doctors’ experiences differed according to care level: while PC doctors experienced better coordination of clinical information, SC doctors experienced greater consistency of care. However, the general perception of patient care coordination within the healthcare area was low in both groups, in contrast to the findings of previous qualitative studies [20]. Factors associated with this perception were mostly interactional and organizational, with differences among levels of care.

### Primary and secondary care doctors experienced high clinical coordination between levels, with direct communication for patient follow-up and accessibility as the major areas needing improvement

In general terms, the clinical information and clinical management coordination experienced across levels of care was high, despite there being several areas for improvement. Doctors reported a relatively high degree of clinical information coordination – higher than in other contexts [19] – which is coherent with various strategies and information coordination mechanisms that have been implemented in Catalonia in recent years, such as EMR or virtual consultations [35, 38]. That said, considering these measures, it is still lower than expected, especially among SC doctors, which might be due to them not yet having developed the habit of regularly using the shared EMR. Moreover, reported access to joint clinical case conferences was particularly low in both care levels, but especially for SC doctors, revealing limited direct communication and feedback between care levels. In fact, lack of communication or direct contact was the main reason for considering care not to be coordinated for both PC and SC doctors. Mutual feedback is crucial for the follow-up of patients, especially in complex cases involving high levels of uncertainty, for which standardization mechanisms such as shared protocols or clinical practice guidelines are less effective [39]. So despite the progress made in the use of health information technology, improvement in direct communication across the care continuum is still needed [30, 40], particularly in situations of high levels of uncertainty.

With regard to clinical management coordination, we should highlight the high level of agreement with the treatments prescribed by the other level and adequateness of referrals and back-referrals, which are consistent with the reported high degree of trust in the clinical skills of doctors of the other level. This reflects the strengths of primary care in the Spanish NHS, in contrast to other healthcare settings where SC doctors have low levels of trust in PC doctors’ clinical skills [19]. Nevertheless, only a small proportion of doctors from both levels of care reported making recommendations and resolving queries with each other regularly, and this limited direct contact seems to also be reflected in PC and SC doctors rarely establishing treatment plans together for complex patients, a result also described in previous qualitative studies [20], suggesting that such decisions are not taken jointly despite the importance and need for it [18, 20, 41].

In terms of accessibility between levels of care, as anticipated and in line with the evidence [20, 42], waiting times for referrals to SC (urgent and standard) were reported to be too long, especially by PC doctors. Long waiting times between levels have implications for quality of care and adequate follow-up by PC doctors [43, 44]. Interestingly enough, while a high proportion of PC doctors reported acceptable waiting times on the patient’s return to PC, half of the SC doctors did not know how long waiting times were for this transition, demonstrating a lack of interest that contradicts expectations for coordinated care provision.

### Interactional and organizational factors are associated with the perception of clinical coordination between levels within the area

Although PC and SC doctors tend to experience high clinical information and clinical management coordination, their general perception of clinical coordination within their healthcare area is relatively low. Their most frequently reported reasons for considering care not to be coordinated were lack of communication or direct contact and lack of coordination mechanisms.

Most factors associated with perceiving high levels of coordination are common to both PC and SC doctors, meaning that such factors could be addressed across the whole healthcare network, regardless of level of care. These factors are mostly interactional and organizational factors, in keeping with the results of other authors [45, 46]. Communication, knowing each other and good relationships between doctors of different care levels are at the core of clinical coordination determinants [18]. In this study, interactional factors associated with perceiving high levels of coordination were: knowing the doctors of the other level, trusting in their clinical skills and believing that their own practice had an influence on the other level, in keeping with studies conducted in other contexts [19] and with relational theory, which underlines knowing each other and mutual respect as key factors for coordination [47].

The positive association of joint clinical case conferences for both levels of care confirms the importance of knowing each other and the need for direct communication mechanisms. In previous qualitative studies [21] clinical case conferences have also emerged as a mechanism that promotes coordination. In fact, the coordination mechanisms found to contribute most to clinical coordination are feedback mechanisms based on mutual adjustment, which include shared EMR, joint clinical case conferences, virtual consultations and telephone [21]. The presence of direct communication coordination mechanisms such as phones, e-mail, or virtual consultations did not emerge as significant associated factors in the statistical models, but were highly emphasised by doctors as a reason for perceiving high levels of clinical coordination, in addition to access to shared EMR.

Besides coordination mechanisms, other organizational conditions are also relevant in improving coordination. A common factor associated with perceiving high levels of clinical coordination for doctors of both levels of care, also pointed out in the scientific literature [48], is for an organization’s management to facilitate coordination, not only by implementing coordination mechanisms but also by creating the adequate conditions for their use, such as ensuring there is enough time available to use them [49], another factor positively associated with the perception of clinical coordination among SC doctors. In fact, lack of time is one of the most frequent reasons given by SC doctors for a low perception of coordination, which is also consistent with the literature [18–21, 50].

Finally, healthcare areas where the same entity manages SC and majority of PC are also positively associated with perceiving a high degree of clinical coordination for doctors of both levels of care. There is evidence suggesting that management integration across the entire care continuum (through aligning economic interests, budget policies, clinical objectives, human resources, etc.) is most likely to promote clinical coordination [18, 46, 51]. Further studies should be performed to comparatively analyse clinical coordination according to management model, particularly in a NHS context.

### Limitations

The main limitation of this study is the potential selection bias resulting from the voluntary, self-administered nature of the questionnaire and having no information on doctors’ reasons for refusing to participate when they did so. However, we can confirm the representativeness of the sample when comparing some characteristics (sex, age, level of care) with the available data on doctors working in the NHS in Catalonia. On the other hand, the online self-administered nature of the questionnaire is also a strength, since it makes it easier to reach doctors and it is simpler, faster and less expensive to conduct than face-to-face surveys [37]. Participation was similar or even higher than reported by other online surveys of doctors [52, 53].

## Conclusions

This is the first study that comprehensively analyses clinical coordination across care levels in a national health system. The application of the COORDENA questionnaire, which can be adapted to other contexts, allowed us to identify fields for improvement in clinical coordination and to provide policy makers with evidence, and could be used to periodically monitor clinical coordination performance in health services.

In Catalonia, in general terms, PC and SC doctors experienced a high level of clinical information and clinical management coordination. The major problems identified related to the lack of direct communication between PC and SC doctors to make recommendations, resolve queries and establish common treatment plans for patients, and limited accessibility between levels of care. This study reveals the important role that mutual feedback and direct communication play in fostering clinical coordination across healthcare levels, both for PC and SC doctors. It is therefore necessary to extend the existing mechanisms or even to implement new ones which enhance mutual feedback between doctors of different levels of care. It is also important to tackle other organizational factors, such as building time into the schedule to use such mechanisms.

Further studies are needed to assess the relevance of coordination mechanisms based on the management model of the area where they are implemented, as well as to explore the difficulties in their use in the Catalan context. Moreover, future research should shed more light on the relationship between high levels of clinical coordination and quality of care.

## Data Availability

The datasets generated and/or analysed in this study are not publicly available because individual privacy could be compromised, but are available from the corresponding author on reasonable request.

## Abbreviations

NHS: National Health System
PC: Primary Care
SC: Secondary Care
EMR: Electronic medical record
HC3: Electronic medical record of Catalonia
OR: Odds Ratio
CI: Confidence Interval
VIF: Variance Inflation Factor
